# Effect of viscous soluble dietary fiber on glucose and lipid metabolism in patients with type 2 diabetes mellitus: a systematic review and meta-analysis on randomized clinical trials

**DOI:** 10.3389/fnut.2023.1253312

**Published:** 2023-08-31

**Authors:** Kun Lu, Tingqing Yu, Xinyi Cao, Hui Xia, Shaokang Wang, Guiju Sun, Liang Chen, Wang Liao

**Affiliations:** ^1^Key Laboratory of Environmental Medicine and Engineering of Ministry of Education, Department of Nutrition and Food Hygiene, School of Public Health, Southeast University, Nanjing, China; ^2^Public Service Platform of South China Sea for R&D Marine Biomedicine Resources, The Marine Biomedical Research Institute, Guangdong Medical University, Zhanjiang, Guangdong, China

**Keywords:** viscous soluble dietary fiber, glucose and lipid metabolism, blood glucose, blood lipid, RCTs, meta-analysis

## Abstract

**Background:**

The effect of viscous soluble dietary fiber on glucose and lipid metabolism in type 2 diabetes mellitus (T2DM) remains controversial, and the dose–response relationship of its effect on blood glucose and blood lipid level is still unclear.

**Methods:**

We conducted comprehensive searches in several databases up to 17 January 2023. We conducted a dose–response analysis of randomized controlled trials (RCTs) to investigate the effect of viscous dietary fiber on glucose and lipid metabolism in patients with T2DM.

**Results:**

Statistical significance was observed in the decreases of glycosylated hemoglobin (HbA1c) (mean difference) [MD = −0.47; 95%CI: (−0.66, −0.27)], fasting blood glucose (FBG) [MD = −0.93; 95%CI: (−1.46, −0.41)], total cholesterol (TC) [MD = −0.33; 95%CI: (−0.46, −0.21)], and low-density lipoprotein and cholesterol (LDL-C) [MD = −0.24; 95%CI: (−0.35, −0.13)]. Contrarily, no difference was observed regarding the level of high-density lipoprotein cholesterol (HDL-C) or triglyceride (TG). In addition, the effect on fasting insulin remains unclear. Results from the subgroup analyses showed that an intervention duration longer than 6 weeks had a significant effect on the HbA1c level; a treatment dosage higher than 8.3 g/day had a significant effect on the FBG level.

**Conclusions:**

Supplementation of viscous dietary fiber is beneficial to control blood glucose and blood lipid in T2DM.

## 1. Introduction

Type 2 diabetes mellitus (T2DM) is a non-communicable chronic disease that is prevalent worldwide. The incidence of T2DM is increasing, posing a heavy burden on the global healthcare system. As estimated, there were 463 million people with diabetes worldwide in 2019, and the number of people with diabetes is estimated to reach 578 million and 700 million by 2030 and 2045, respectively (1). The main characteristic of T2DM is glucose metabolism disorder. In addition, it is often accompanied by other metabolic disorders, such as obesity, hyperlipidemia, hypertension, and kidney disease, which lead to neuropathy, as well as microvascular and macrovascular complications. Therefore, it is of great significance to control and manage blood glucose and blood lipid levels in patients with T2DM to prevent its potential complications.

Nutritional intervention is one of the key approaches to managing T2DM (2, 3). Notably, dietary fiber could contribute to improving gastrointestinal health, which could further impact lipid metabolism (4). Dietary fiber intake has been linked to a lower risk of T2DM (5). Dietary fiber is classified as soluble or insoluble, based on its solubility in hot water (6). It has been suggested that soluble dietary fiber could reduce energy intake and delay the hydrolysis and absorption of nutrients in the small intestine by increasing satiety (7, 8). It has also been reported that soluble dietary fiber could improve glucose metabolism and lipid distribution in patients with type 2 diabetes (9–11). However, some studies reported contradictory results (12–14). Soluble fiber can be divided into non-viscous fiber and viscous fiber, according to its viscosity. Major types of viscous fiber include psyllium, guar gum, β-glucan, glucomannan, and Cassia tora (6). Previous studies have shown that some highly viscous soluble fibers, such as guar gum, psyllium, and β-glucan, have a significant effect on lowering blood glucose or glycemic index (GI), and the effect is positively correlated with viscosity (15). The underlying mechanism was thought to be the water-holding ability of viscous fibers, which can form a gel matrix and slow down gastric emptying. Simultaneously, this gel matrix thickens the small intestinal contents, slows down the small intestinal transit time, and reduces the contact of nutrients with digestive enzymes, thereby reducing blood glucose levels (16). However, other studies have reached inconsistent conclusions. A study investigating the effect of cereal fiber and fruit fiber on type 2 diabetes (6) suggested that sticky soluble dietary fiber (from fruit sources) had a weak protective effect on the risk of T2DM, and this conclusion was based on a cohort study. In this meta-analysis, we selected RCTs, which are generally more controllable and have a higher level of evidence than cohort studies. In addition, some RCTs also reported consistent results, suggesting that sticky soluble dietary fiber has no effect on glucose and lipid metabolism (17–21). The benefits of sticky fiber in people with T2DM are controversial. This may be related to the experimental design, small sample size, insufficient dose, and other factors. Therefore, we conducted a meta-analysis to expand the sample size by including recent studies, aiming to draw a solid conclusion.

## 2. Materials and methods

The study followed the 2020 Preferred Reporting Items for Systematic Reviews and Meta-analysis (PRISMA) guidelines.

### 2.1. Literature search strategy

We systematically searched for relevant articles published until 17 January 2023: PubMed, Web of Science, Embase, and Cochrane Library. The keywords were related to the research objectives. In our search strategy, we combined MeSH and non-mesh keywords without date or language restrictions. The search terms were as follows: (diabetes mellitus, type 2 OR type 2 diabetes OR diabetes mellitus, type II OR type 2 diabetes mellitus OR type 2 diabetes OR diabetes, type 2 OR diabetes mellitus, non-insulin dependent OR NIDDM OR diabetes mellitus, non-insulin dependent OR non-insulin-dependent diabetes mellitus OR non-insulin dependent diabetes mellitus OR diabetes mellitus, maturity onset OR diabetes mellitus, and adult onset OR T2DM or DMT2); (dietary fiber OR dietary fibers OR fibers, dietary OR fiber, and dietary); and (randomized controlled trial OR randomized controlled trial OR controlled clinical trial OR clinical trial, randomized OR randomized, and trial OR randomized OR intervention OR controlled trial OR random OR placebo). In addition, we manually searched the reference lists of each study to supplement relevant studies that may have been overlooked.

### 2.2. Inclusion and exclusion criteria

This study strictly included original research according to the following criteria: (1) the study was an RCT with a parallel or crossover design with an experimental period of more than 2 weeks; (2) the study subjects were adults with T2DM (aged ≥ 18 years); (3) the intervention of interest was the supplementation of viscous soluble dietary fiber (such as psyllium, guar gum, β-glucan, glucomannan, Cassia tora, and other viscous soluble dietary fibers), with a placebo, insoluble fiber, or without fiber supplementation in the control group; (4) the outcome indicators included at least one blood glucose control indicator (HbA1c, fasting blood glucose, or fasting insulin) and one blood lipid control indicator (TC, LDL-C, HDL-C, or TG); and (5) the data were complete and could provide the basis for subsequent analyses.

We excluded literature based on the following criteria: (1) studies with subjects who had type I diabetes, gestational diabetes, or metabolic syndrome; studies with subjects who were adolescents or children; (2) experiments with a study period that was too short (< 2 weeks); (3) studies that combined interventions or could not separate the effect of viscous soluble dietary fiber on blood glucose or blood lipids; (4) cytological studies, animal experiments, non-controlled trials, or non-clinical studies; and (5) trials with incomplete or irrelevant data.

### 2.3. Data extraction

Two researchers independently evaluated the included literature and extracted the following data by reading the full text: name of the first author, year of publication, types of study design, country, sample capacity, gender, average age of participants, BMI, duration of diabetes, type of fiber in the experimental group, substance in the control group, daily intake of viscous soluble dietary fiber, and cycles of intervention. In addition, the mean and standard deviation of blood glucose control indicators (HbA1c, FBG, or fasting insulin) and blood lipid control indicators (TC, LDL-C, HDL-C, or TG) in each literature were extracted. If not reported, they were converted based on available data (95% CIs, SEM, or median).

### 2.4. Quality assessment of studies

The literature included in the study was evaluated for risk bias using the Cochrane Bias Risk Tool, which assesses seven validity questions as follows: random sequence generation (selection bias), allocation concealment (selection bias), blinding of participants and personnel (performance bias), blinding of outcome assessment (detection bias), incomplete outcome data (attrition bias), selective reporting (reporting bias), and other bias. The bias risk of each validity question is divided into low risk, high risk, and unclear risk.

### 2.5. Statistical analysis

Excel 2010 was used to collect and organize literature data and to standardize the different units of blood glucose and blood lipid levels: FBG was converted to mmol/L (18.0 mg/dL = 1 mmol/L), fasting insulin was converted to μIU/mL (1 μIU/mL = 6.00 pmol/L, 1 mU/L = 1 μIU/mL) (22), and blood lipid units were converted to mmol/L (TC, LDL-C, HDL-C: 1 mmol/L = 1 mg/dL × 0.02586, TG:1 mmol/L = 1 mg/dL × 0.01129). Subsequent data were analyzed using Review Manager 5.4 and Stata 15.1. Mean differences (MD) and standard deviations (SD) of each outcome variable before and after intervention were calculated using the formula: SD_change_ = (SD${}_{\text{baseline}}^{2}$ + SD${}_{\text{endpoint}}^{2}$ – 2R × SD_baseline_ × SD_endpoint_) 1/2, with a correlation coefficient R = 0.5 (23). The heterogeneity of the studies was evaluated by conducting a chi-square test and using the I^2^ index. When the *p*-value of the chi-square test was < 0.10 or I^2^ was >50%, significant heterogeneity among the studies was considered to exist, and a random-effects model was used. Subgroup analysis was conducted based on region, study type, fiber type, dosage, and duration to explore possible sources of heterogeneity. Sensitivity analysis was performed by removing one or two studies to assess the stability of the overall statistical results. In addition, this study used a funnel plot and Egger's regression test to evaluate possible publication bias. Meta-regression was used for dose–response analysis.

## 3. Results

### 3.1. Literature search and study characteristics

**Figure 1:**
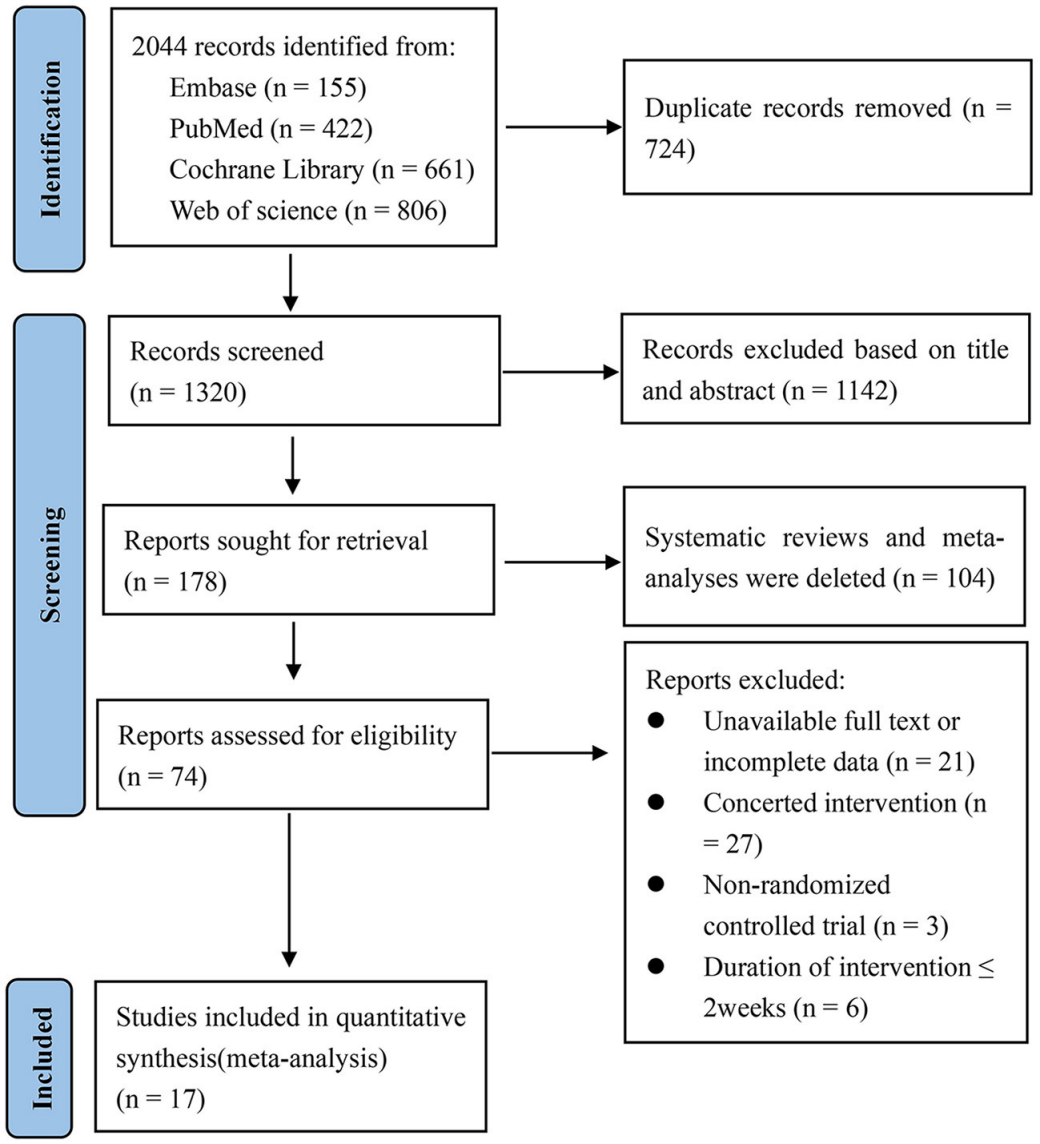
Flow chart of literature selection.

The specific process of literature retrieval and screening is shown in Figure 1. According to the search strategy, 2,044 studies were retrieved from the four databases; 724 duplicated studies were removed and 1,246 irrelevant studies were excluded based on the title and abstract, of which 104 were systematic reviews and meta-analyses. Then, the remaining 74 studies were evaluated as a whole. Among them, 57 studies were excluded due to the following reasons: unavailability of the full text or incomplete data, combined intervention or inability to separate the effect of viscous soluble dietary fiber on blood glucose or blood lipids, non-randomized controlled trials, and short intervention period. Finally, 17 studies (24–40) met the inclusion criteria (10 parallel studies and 7 crossover studies). It should be noted that in two studies the data were divided into two groups. In one study, we selected the data at the end of week 16, while in other studies, we extracted the data at the end of the study. Therefore, we finally included a total of 19 datasets in the meta-analysis.

### 3.2. Study characteristics and risk of bias assessment

The characteristics of each study are shown in Supplementary Table 1. Among the included studies, 4 studies were conducted in North America (Canada-1 and America-3), and 5 studies were conducted in Asia (China-1, Korea-1, Palestine-1, and Iran-2). Ten studies were conducted in Europe (France-1, Greece-1, Italy-1, Finland-3, and the UK-4). A total of 642 participants were included in the studies, with a mean age of 51.9 to 66.5 years. The duration of diabetes was stable for more than 1 year in all the studies, except for one where diabetes was newly diagnosed and another with the duration unknown. The supplementation dose of viscous soluble dietary fiber ranged from 3 to 21 g/day, and the supplementation period was from 3 to 16 weeks.

The risk of bias assessment of the included studies is shown in Table 1. Most studies were considered to have a low risk of bias, while random sequence generation and other biases were unknown because not enough information is available for risk rating.

**Table 1:** Risk of bias assessment.

**Study**	**Random sequence generation**	**Allocation concealment**	**Blinding of participants and personnel**	**Blinding of outcome assessment**	**Incomplete outcome data**	**Selective reporting**	**Other bias**
Anderson et al. (25)	U	L	L	L	L	L	U
Aro et al. (26)	U	L	L	L	L	L	U
Abutair et al. (27)	U	U	H	U	L	L	U
Chen et al. (28)	U	L	L	L	L	L	U
Cho et al. (29)	U	L	L	L	L	L	U
Cugnet-Anceau et al. (24)	U	L	L	L	L	L	U
Feinglos et al. (40)	U	L	L	L	L	L	U
Fuessl et al. (30)	U	L	L	L	L	L	U
Ghalandari et al. (31)	U	L	L	L	L	L	U
Lalor et al. (32)	U	L	L	L	L	L	U
Liatis et al. (33)	L	L	L	L	L	L	U
Niemi et al. (34)	U	L	L	L	L	L	U
Peterson et al. (35)	U	U	H	U	L	L	U
Reimer et al. (36)	U	L	L	L	L	L	U
Uusitupa et al. (37)	U	U	H	U	L	L	U
Vuksan et al. (38)	U	L	L	L	L	L	U
Ziai et al. (39)	U	L	L	L	L	L	U

L, low; H, high; U, unclear.

### 3.3. Results of meta-analysis

**Figure 2:**
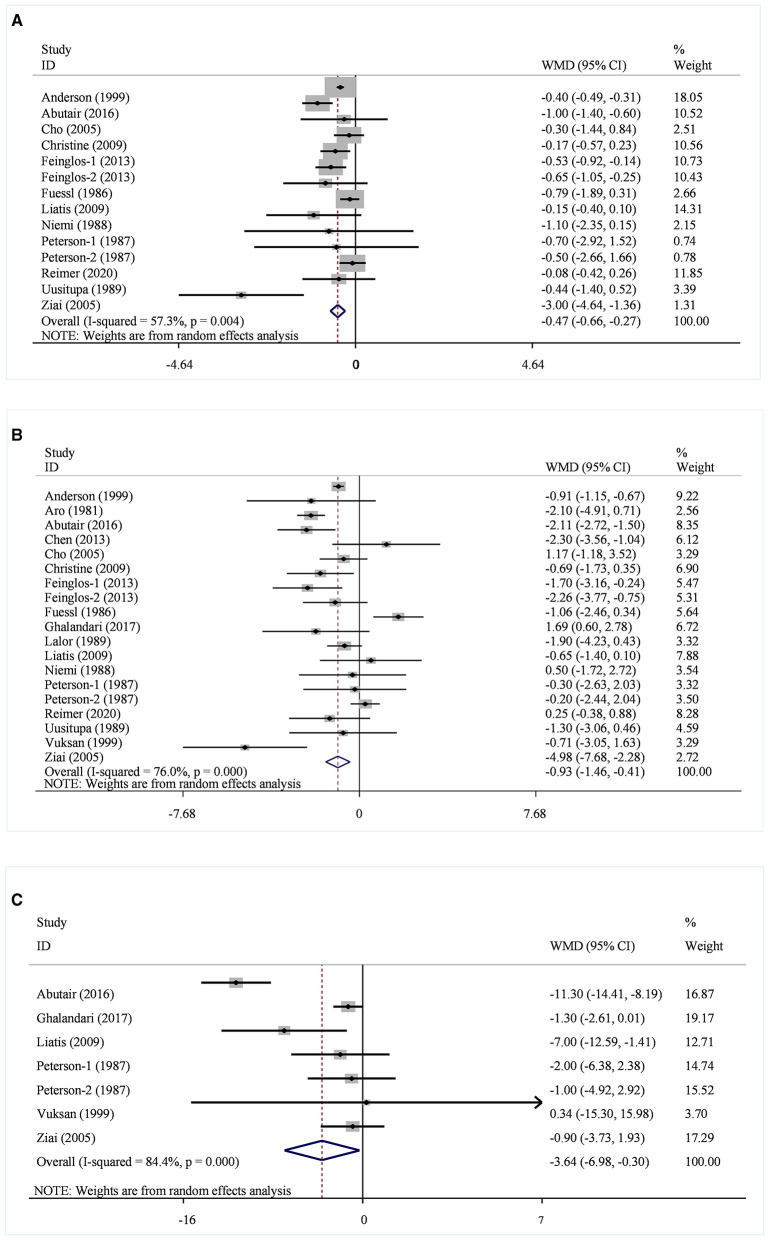
Effects of viscous soluble dietary fiber on HbA1c **(A)**, FBG **(B)**, and fasting insulin **(C)**. HbA1c, glycosylated hemoglobin; FBG, fasting blood glucose; WMD, weighted mean difference.

**Figure 3:**
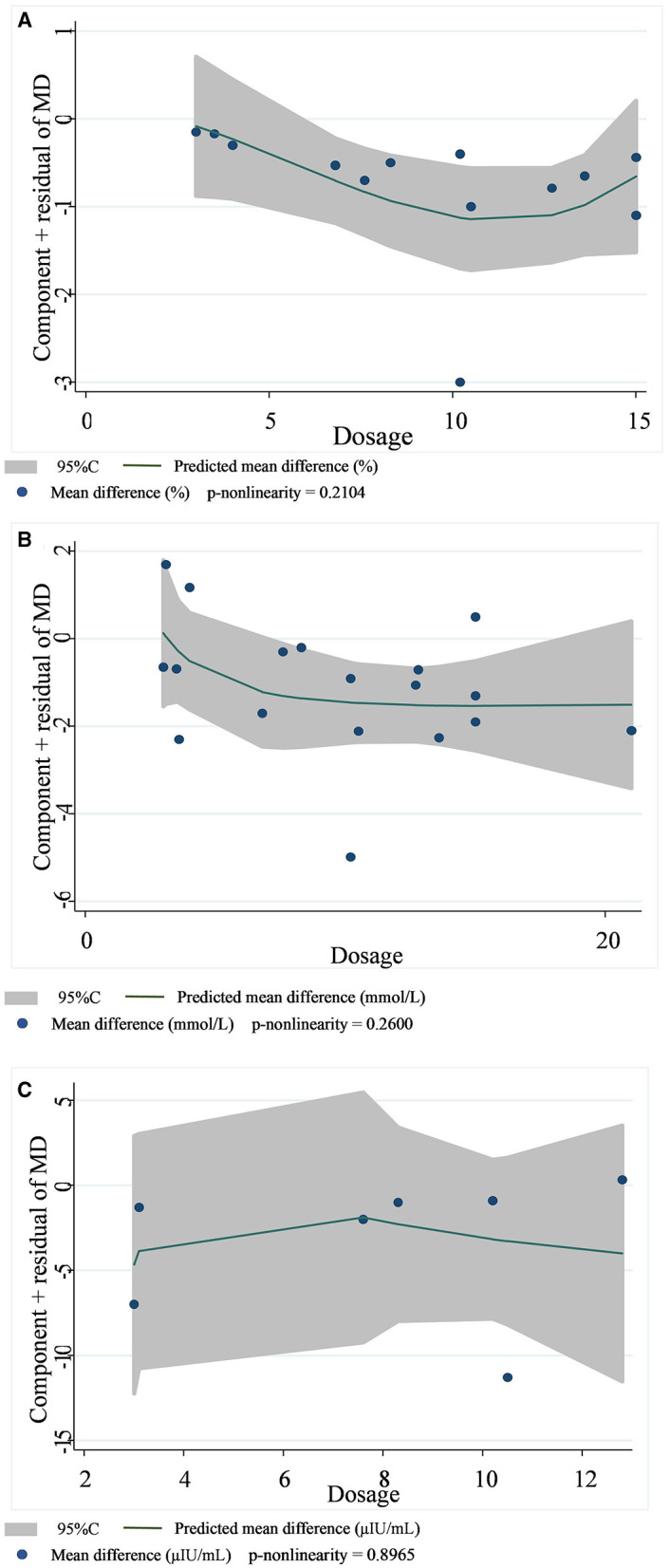
Non-linear dose–response relationships between viscous soluble dietary fiber (g) and the nonstandard mean difference (g/day) in HbA1c **(A)**, FBG **(B)**, and fasting insulin **(C)**. 95%CI is displayed in shaded areas. HbA1c, glycosylated hemoglobin; FBG, fasting blood glucose.

**Figure 4:**
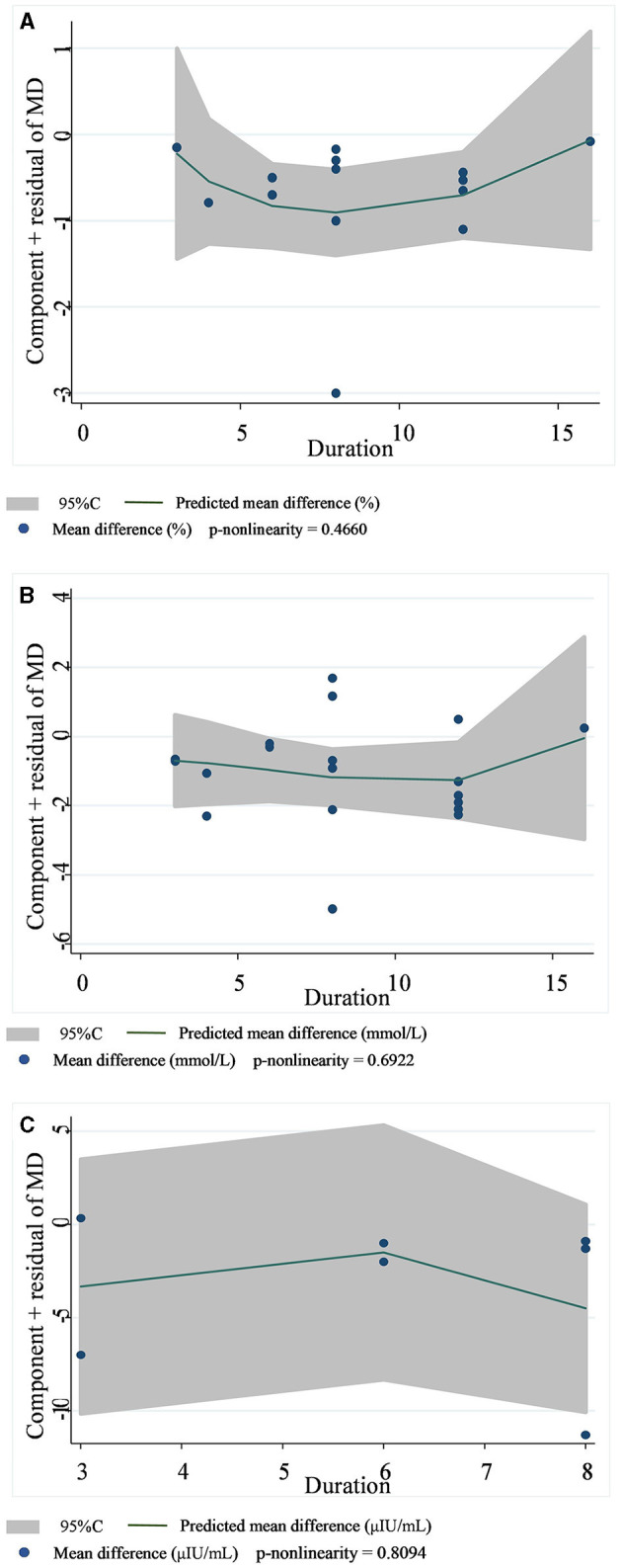
Non-linear dose–response relationships between supplementation duration (weeks) and the nonstandard mean difference (g/day) in HbA1c **(A)**, FBG **(B)**, and fasting insulin **(C)**. 95%CI is displayed in shaded areas. HbA1c, glycosylated hemoglobin; FBG, fasting blood glucose.

A total of 14 datasets from 12 studies were included in the HbA1c analysis, and the random effect model showed that the supplementation of viscous soluble dietary fiber could significantly reduce the HbA1c level [MD = −0.47; 95%CI: (−0.66, −0.27), *p* < 0.001, I^2^ = 57.3%, *p* = 0.004, Figure 2A]. In addition, in the non-linear dose–response analysis, we did not observe a significant effect of the dosage of viscous soluble dietary fiber supplementation on HbA1c (p-non-linearity = 0.2104, Figure 3A). Furthermore, no significant effect of the supplementation period on HbA1c was observed (p-non-linearity = 0.4660, Figure 4A).

Based on the 19 datasets from 17 studies, the reduction of FBG by viscous soluble dietary fiber was found to be statistically significant [MD = −0.93; 95%CI: (−1.46, −0.41), *p* = 0.001, I^2^ = 76.0%, *p* < 0.001, Figure 2B]. However, we found an insignificant non-linear relationship between FBG and supplementation dose (p-non-linearity = 0.2600, Figure 3B) and the supplementation period (p-non-linearity = 0.6922, Figure 4B) of viscous soluble dietary fiber.

Across 7 datasets from 6 studies, we found a significant effect of viscous soluble dietary fiber on fasting insulin under the random-effects model [MD = −3.64; 95%CI: (−6.98, −0.30), *p* = 0.033, I^2^ = 84.4%, *p* < 0.001, Figure 2C]. The non-linear dose–response relationship between fasting insulin and supplementation dose (p-non-linearity = 0.8965, Figure 3C) and supplementation period (p-non-linearity = 0.8094, Figure 4C) was not statistically significant.

**Figure 5:**
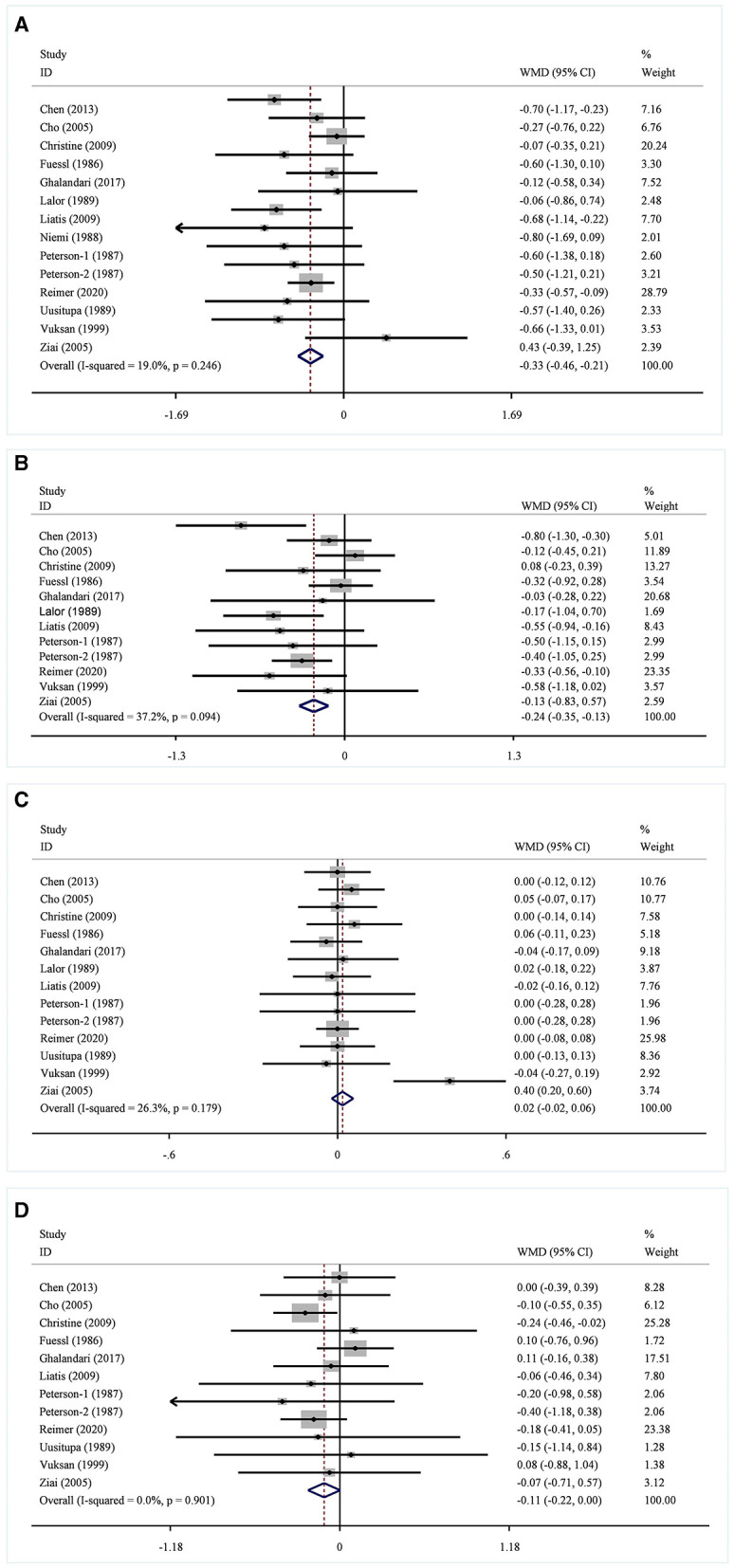
Effects of viscous soluble dietary fiber on TC **(A)**, LDL-C **(B)**, HDL-C **(C)**, and TG **(D)**. TC, total cholesterol; LDL-C, low-density lipoprotein cholesterol; HDL-C, high-density lipoprotein cholesterol; TG, triglyceride; WMD, weighted mean difference.

**Figure 6:**
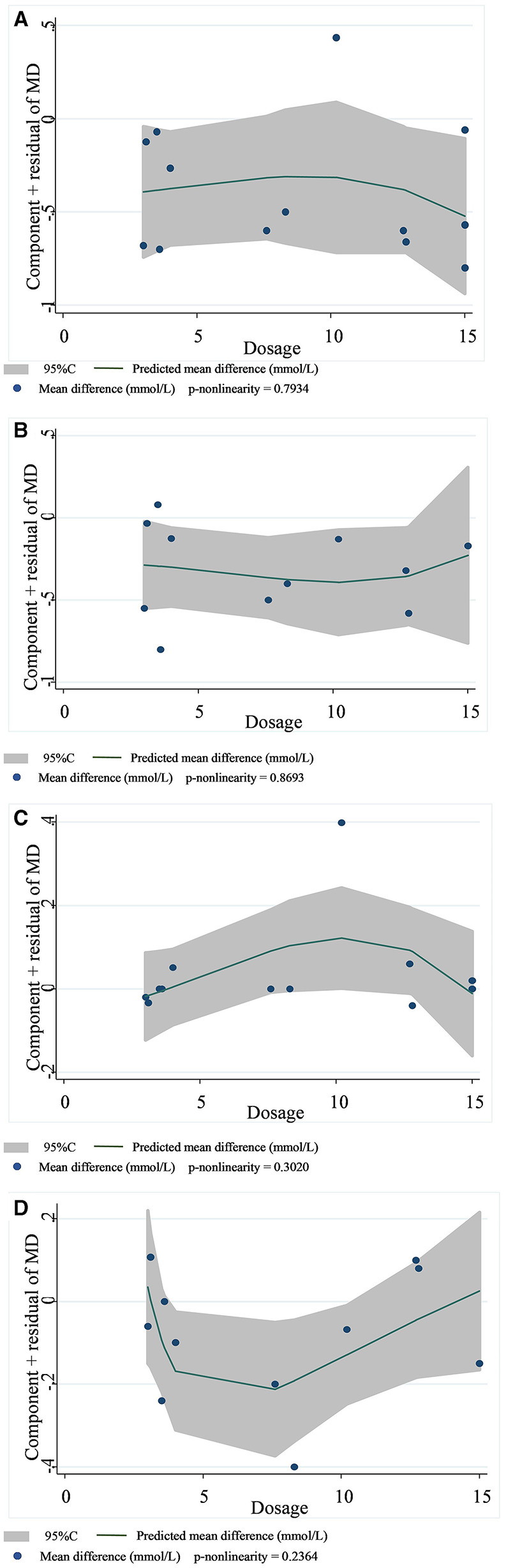
Non-linear dose–response relationships between viscous soluble dietary fiber (g) and the nonstandard mean difference (g/day) in TC **(A)**, LDL-C **(B)**, HDL-C **(C)**, and TG **(D)**. 95%CI is displayed in shaded areas. TC, total cholesterol; LDL-C, low-density lipoprotein cholesterol; HDL-C, high-density lipoprotein cholesterol; TG, triglyceride.

**Figure 7:**
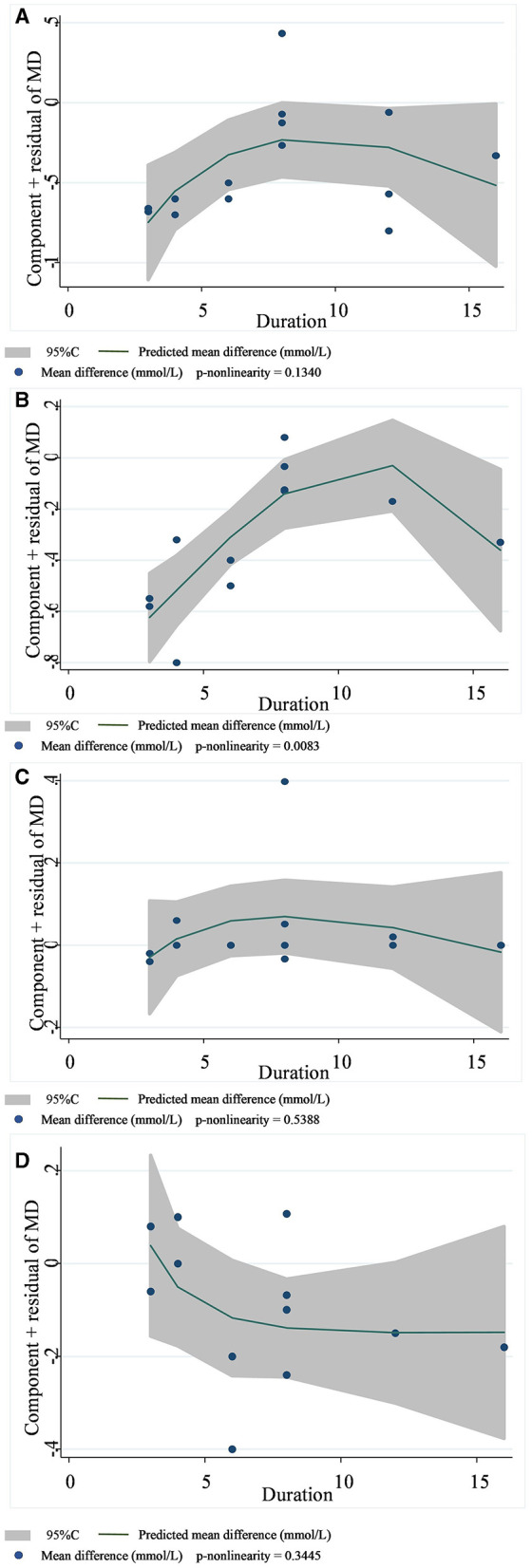
Non-linear dose–response relationships between supplementation duration (weeks) and the nonstandard mean difference (g/day) in TC **(A)**, LDL-C **(B)**, HDL-C **(C)**, and TG **(D)**. 95%CI is displayed in shaded areas. TC, total cholesterol; LDL-C, low-density lipoprotein cholesterol; HDL-C, high-density lipoprotein cholesterol; TG, triglyceride.

In 13 studies of 14 groups of data analyzed for the TC level, the effect of viscous soluble dietary fiber on the TC level was statistically significant [MD = −0.33; 95%CI: (−0.46, −0.21), *p* < 0.001, I^2^ = 19.0%, *p* = 0.246, Figure 5A]. However, in the non-linear dose–response analysis, the supplementation dose (p-non-linearity = 0.7934, Figure 6A) and supplementation period (p-non-linearity = 0.1340, Figure 7A) had no significant effect on the TC level.

In the LDL-C analysis, we found that viscous soluble dietary fiber had a significant effect on the reduction of LDL-C from 12 datasets obtained from 11 studies [MD = −0.24; 95%CI: (−0.35, −0.13), *p* < 0.001, I^2^ = 37.2%, *p* = 0.094, Figure 5B]. In addition, we failed to find a significant relationship in the non-linear dose–response relationship between LDL-C and the dosage of viscous soluble dietary fiber supplementation (p-non-linearity = 0.8693, Figure 6B). However, we observed a statistically significant non-linear relationship between LDL-C and the supplementation period (p-non-linearity = 0.0083, Figure 7B).

As for the effect variable of HDL-C, 13 groups of data from 12 studies were included in the analysis. According to the analysis results, the effect of viscous soluble dietary fiber on HDL-C was statistically significant [MD = 0.02; 95%CI: (−0.02, 0.06), *p* = 0.367, I^2^ = 26.3%, *p* = 0.179, Figure 5C]. In addition, we found that concerning the non-linear dose–response relationship analysis, the effect of the supplementation dose (p-non-linearity = 0.3020, Figure 6C) and supplement period (p-non-linearity = 0.5388, Figure 7C) on HDL-C was not statistically significant.

The analysis of the TG level was based on 12 groups of datasets from 11 studies. We failed to observe a significant effect of viscous soluble dietary fiber on the TG level [MD = −0.11; 95%CI: (−0.22, 0.00), *p* = 0.060, I^2^ = 0.0%, *p* = 0.901, Figure 5D]. Similarly, we also found that neither the supplementation dose (p-non-linearity = 0.2364, Figure 6D) nor the supplementation period (p-non-linearity = 0.3445, Figure 7D) had a non-linear dose–response relationship with the TG level.

### 3.4. Subgroup analysis

The heterogeneity of HbA1c, FBG, and fasting insulin was observed to exceed 50%, indicating significant heterogeneity. Therefore, a subgroup analysis was conducted, and the results are shown in Supplementary Table 2.

The subgroup analysis results showed that the heterogeneity of HbA1c between studies disappeared when grouped by region (I^2^ = 0.0%, *p* = 0.712), study type (I^2^ = 0.0%, *p* = 0.963), fiber type (I^2^ = 0.0%, *p* = 0.947), dose of viscous soluble dietary fiber (I^2^ = 0.0%, *p* = 0.706), and intervention period (I^2^ = 0.0%, *p* = 0.679). From these analyses, we observed significant changes in HbA1c in T2DM subjects in trials in which the fiber type was psyllium [MD = −0.72; 95%CI: (−1.08, −0.37), *p* < 0.001] or guar gum [MD = −0.70; 95%CI: (−1.28, −0.12), *p* = 0.018] and those in which the duration of viscous dietary fiber was >6 weeks [MD = −0.52; 95%CI: (−0.76, −0.28), *p* < 0.001].

For FBG, the inter-study heterogeneity disappeared when subgroup analysis was performed by the study region (I^2^ = 0.0%, *p* = 0.907), study type (I^2^ = 6.4%, *p* = 0.381), fiber type (I^2^ = 0.0%, *p* = 0.701), and the intervention period of viscous soluble dietary fiber (I^2^ = 14.0%, *p* = 0.325). It remained high when subgroup analysis was performed by supplementation dosage (I^2^ = 75.3% or 76.4%, *p* < 0.001). In addition, viscous soluble dietary fiber was effective in reducing FBG in trials conducted in North America [MD = −0.94; 95%CI: (−1.83, −0.05), *p* = 0.038] and Europe [MD = −0.76; 95%CI: (−1.22, −0.29), *p* = 0.001], in trials with β-glucan [MD = −0.66; 95%CI: (−1.27, −0.05), *p* = 0.033], psyllium [MD = −1.40; 95%CI: (−2.50, −0.31), *p* = 0.012], and glucomannan [MD = −1.82; 95%CI: (−3.25, −0.40), *p* = 0.012] or guar gum [MD = −0.91; 95%CI: (−1.67, −0.15), *p* = 0.020], and in trials with doses >8.3 g/day [MD = −1.29; 95%CI: (−1.97, −0.61), *p* < 0.001].

Additionally, the study heterogeneity also disappeared in subgroup analyses concerning fasting insulin. In parallel trials [MD = −4.93; 95%CI: (−9.84, −0.02), *p* = 0.049] or in trials with supplementation doses ≤ 10.2 g/day [MD = −1.49; 95%CI: (−2.62, −0.36), *p* = 0.010], the effect of viscous soluble dietary fiber supplementation was significant in T2DM subjects.

### 3.5. Sensitivity analysis

Sensitivity analysis was performed, excluding one or two studies at a time to observe their impact on the overall results. The results are shown in Supplementary Table 3. For HbA1c, studies of Abutair et al. (27) and Ziai et al. (39) were excluded, and the overall heterogeneity was changed (from I^2^ = 57%, *p* = 0.004 to I^2^ = 4%, *p* = 0.40), but the effect variable only increased by 0.11%. After eliminating the data from Abutair et al. study (27), the overall heterogeneity of fasting insulin changed (from I^2^ = 84%, *p* < 0.00001 to I^2^ = 0%, *p* = 0.53). The effect variables also changed [from MD = −3.64; 95% CI: (6.98, 0.30), *p* < 0.00001 to MD = 1.47; 95%CI: (−2.55, −0.39), *p* = 0.008], increasing by 2.17. After excluding the studies of Cugnet-Anceau et al. (24) and Ziai et al. (39), the overall heterogeneity of TC also changed. When Cugnet-Anceau et al. study (24) was removed, the overall heterogeneity of TC changed (from I^2^ = 19%, *p* = 0.0.25 to I^2^ = 0%, *p* = 0.46). Similarly, when Ziai et al. study was removed (39), the overall heterogeneity of TC was reduced to (I^2^ = 5%, *p* = 0.40). However, when these two studies were deleted separately, the overall effect variable of TC barely changed. For LDL-C, after removing the studies of Chen et al. (28) and Cugnet-Anceau et al. (24), the overall heterogeneity changed (from I^2^ = 37%, *p* = 0.09 to I^2^ = 0%, *p* = 0.48), but the change in the overall effect variable could be ignored. Similarly, after removing the study conducted by Ziai et al. (39), the overall heterogeneity of HDL-C was reduced (from I^2^ = 26%, *p* = 0.18 to I^2^ = 0%, *p* = 1.00), and the overall effect variable was almost negligible. For FBG and TG, there was no significant change in heterogeneity or population effect variables after deleting any studies.

### 3.6. Publication bias analysis

The publication bias of HbA1c, FBG, fasting insulin, TC, LDL-C, HDL-C, and TG was initially assessed by funnel plot, and no significant asymmetry was found (Supplementary Figure). In addition, the Egger test results showed that the *p*-values of HbA1c: *p* = 0.236; FBG: *p* = 0.870; fasting insulin: *p* = 0.502; TC: *p* = 0.315; LDL-C: *p* = 0.190; HDL-C: *p* = 0.382; and TG: *p* = 0.652 were all greater than 0.05, and there was no evidence of potential publication bias in the combined results.

## 4. Discussion

Although a large number of studies have reported the beneficial effects of dietary fiber in the human body, the effect was actually associated with the type of dietary fiber (41). This systematic review aimed to investigate the effect of sticky soluble dietary fiber on glucose and lipid metabolism in patients with T2DM. Seventeen RCTs were included, and we found that sticky soluble dietary fiber can significantly modulate the levels of HbA1c, FBG, and fasting insulin in patients with T2DM. The effect on the TC and LDL-C levels in patients was also significant. Furthermore, the analysis of the non-linear dose–response relationship revealed a correlation between the supplementation cycle and LDL-C levels.

Although previous meta-analyses have shown that some sticky soluble dietary fibers are not effective in controlling certain glycemic indices (17, 19, 21, 42, 43), we collected and included recent studies and found that viscous soluble dietary fiber supplementation had a significant effect on HbA1c, FBG, and fasting insulin levels in patients with T2DM. In our study, the average total effect of HbA1c was reduced by 0.47% in patients with T2DM. A study on the acute effect of soluble dietary fiber on postprandial blood glucose in T2DM showed that soluble dietary fiber supplementation could reduce postprandial blood glucose, and postprandial blood glucose level contributed to HbA1c (44). HbA1c accurately reflects long-term glycemic control. Studies have shown that a 1% reduction in HbA1c significantly reduces the risk of complications associated with T2DM, such as peripheral vascular disease, microvascular complications, myocardial infarction, and stroke (45). Another meta-analysis of a prospective cohort study revealed an association between HbA1c levels and cardiovascular risk in patients with diabetes, with increased cardiovascular risk associated with increased HbA1c levels (46). Moreover, a recent 6-year follow-up study suggested that early control of HbA1c levels in newly diagnosed T2DM patients is more conducive to long-term and lasting glycemic control than late control, and it can better reduce the incidence of diabetes complications, especially microvascular complications (47). This suggests the importance of controlling HbA1c levels for diabetic patients. In our subgroup analysis, the effect of viscous fibers on HbA1c was significant only when the duration of supplementation was longer than 6 weeks, which is consistent with a previous study that demonstrated a reduction in HbA1c levels at weeks 4 and 6 with guar gum supplementation (19).

Similarly, FBG is also an important indicator for evaluating the effectiveness of blood glucose control. Previous studies have shown that sticky soluble dietary fiber can significantly reduce FBG levels in patients with T2DM (48, 49). In addition, a meta-analysis showed that 12 g/day of psyllium fiber reduced FBG by an average of 37 mg/dL (2.06 mmol/L) in patients with T2DM (50). In our study, the overall effect of viscous soluble fiber on the reduction of FBG was 0.93 mmol/L, and the difference in values may be related to the lower dose of the fiber intake (the median dose included in this study was 10.2 g/day). It is important to note that in the subgroup analysis we performed, viscous soluble dietary fiber did not have a significant effect on the reduction of FBG when the dose was ≤ 8.3 g/day, and the inclusion of such studies in our meta-analysis may have led to an underestimate of its benefit. However, our results were inconsistent with a previous report where daily supplementation of 7.6–8.3 g of soluble dietary fiber could effectively control the blood sugar of patients with type 2 diabetes and improve insulin resistance (51); the inclusion of non-viscous fiber might be the underlying reason for the difference. In addition, the difference in values may also be due to the fact that this previous study (50) only used psyllium fiber, while there were a variety of viscous fibers involved in our study. In our subgroup analysis, the type of fiber imposed a significant impact on the reduction of FBG (Supplementary Table 2). The viscosity and the stickiness of the fiber may have contributed to such a difference (15). The chemical structure of different types of viscous fibers is different, resulting in differences in their viscosity and specific functions in the gastrointestinal tract. β-glucans are composed of glucose molecules connected by a β-glycosidic bond (7). The viscosity of β-glucans with the same molecular weight but different volumes is different, but there is no significant difference in the effect on blood glucose. The objective existence and high molecular weight of β-glucans are more important than their volume in regulating blood glucose; the viscosity of the digesta produced after consumption in response to amylase is responsible for gastric emptying and glucose absorption, rather than the initial concentration of the fiber solution (16). The mucopolysaccharide mixture of psyllium, which is composed of pentose, hexose, and uronic acids, cannot be fermented in the body; it maintains a persistent water-holding capacity and swelling effect in the intestine (7), inhibits glucose diffusion, α-amylase, and pancreatic lipase activities, lowers postprandial blood glucose and lipid levels, and binds to bile acids to reduce cholesterol (52). Guar gum and glucomannan are fermentable fibers. In addition to increasing the viscosity of small intestinal contents and affecting gastric emptying, they can also produce beneficial products through fermentation in the colon, mainly short-chain fatty acids (SCFAs) such as propionate, which can indirectly inhibit the biosynthesis of cholesterol and fatty acids (53, 54).

Patients with T2DM are often accompanied by insulin resistance, and the fasting insulin level is positively correlated with insulin resistance (55). A cohort study conducted in the Netherlands found a linear relationship between fasting insulin and the incidence of T2DM, with lower fasting insulin levels associated with lower risk (56). Another study showed a stronger association, suggesting that people with high fasting insulin levels were more likely to develop T2DM (57). Our study found that increasing viscous dietary fiber intake improved fasting insulin levels in patients with T2DM, which is consistent with the findings of other studies (20, 42, 58). However, the results of another meta-analysis (49) showed that β-glucan did not improve fasting plasma insulin concentration in subjects with T2DM. Interestingly, the subgroup analysis results in this study showed that the effect of viscous dietary fiber was not significant, except for the dosage ≤ 10.2 g/day and for the β-glucan fiber type. However, only one study with a β-glucan fiber type was included in our meta-analysis. Therefore, more long-term and high-quality RCTs in this aspect are needed.

Previously, it was found that long-term supplementation of medium to high doses of sticky soluble fiber (psyllium and guar gum) improved metabolic indices in patients with metabolic syndrome. By the fourth month of intervention, the reduction in FBG and LDL-C ranged from 9.3 to 16.4% and from 4.3 to 4.4%, respectively. The beneficial effect was more obvious by the 6th month of intervention, with the reduction range of FBG increasing to 11.1–27.9%, that of LDL-C decreasing by 7.9–8.5%, and that of TC decreasing by 6.3–7.5%; by the 6th month, psyllium supplement improved TG concentration significantly (−13.3%) (59). The effect of improving the lipid profile of soluble dietary fiber was also demonstrated by another meta-analysis of guar gum, where guar gum significantly reduced TC and LDL-C, but the intervention did not change the TG or HDL-C levels (60). However, the above studies were not restricted to patients with T2DM. In our results, sticky fibers significantly reduced the TC and LDL-C levels in patients with T2DM, and the heterogeneity among these studies was low. Additionally, we performed a dose–response analysis and found a non-linear relationship between the intervention period of sticky soluble dietary fiber and LDL-C levels, suggesting that the length of the period affects the effect of sticky fiber on LDL-C. When the intervention period was < 12 weeks, the reduction level of viscous soluble dietary fiber on LDL-C was better, with the extension of the intervention time, but when the intervention period was more than 12 weeks, the effect showed a reverse trend. Such a phenomenon is in contradiction with previous findings (59) that the LDL-C reduction effect is more significant when supplementing with viscous fiber for 6 months than when supplementing with it for 4 months. We think the possible reasons for this are as follows: many types of viscous fibers were involved in our study, and different fiber types may have affected the results. The longest study period in our study was 16 weeks; considering that Cicero et al. (59) studied up to 6 months, our existing results may change when studies with longer periods are included. Hence, we recommend longer RCTs to find regular changes in glycemic and lipid improvement during intervention with sticky soluble dietary fiber.

Due to the high heterogeneity between studies, subgroup analysis of HbA1c, FBG, and fasting insulin was performed. Except for the dose in the FBG index, the heterogeneity among the other subgroups changed significantly. According to the comprehensive results, all the factors for subgroup analysis may be the sources of heterogeneity. To further determine the source of heterogeneity, we also conducted a meta-regression analysis. Accordingly, the region and fiber type were confirmed as potential sources of heterogeneity in the regression of HbA1c. Furthermore, sensitivity analysis was conducted to evaluate the stability of the results to exclude studies that affected the heterogeneity. We found that removing the study conducted by Ziai et al. (39) significantly affected the heterogeneity of the studies. We reviewed the study but found no probable factors contributing to the heterogeneity, and the bias risk assessment tool acknowledged the high quality of this study. Finally, by comparing the original association results with the association results after removing the studies that significantly affected the heterogeneity, we found that the overall results before and after the removal did not change significantly. In other words, although these studies affected the overall heterogeneity of the study, our results were still stable.

Although some previous studies have suggested that sticky soluble dietary fiber is beneficial for glycemic and lipid control in patients with T2DM, this meta-analysis synthesized and quantified the effect of sticky soluble dietary fiber on adults with T2DM. Additionally, a dose–response analysis was performed to investigate the effect of supplemental dose and intervention period on the efficacy of sticky dietary fiber. Furthermore, the studies we included were across several ethnic and geographic groups, which enhanced the generalizability of the results. In this meta-analysis, the risk of bias was considered to be low, and the results were evaluated objectively, which provided some reliability for the final results and conclusions.

Results from the present meta-analysis suggested that viscous soluble dietary fiber can be used as a dietary supplement for the management of T2DM. However, it should be noted that the high viscosity of viscous fiber can lead to excessive viscosity during swallowing and reduce the palatability of food. Hence, the development of palatable viscous fiber foods is still a challenge. At present, the commonly used food processing method involves adding viscous fibers to proteins, starches, or beverages or adding acidic fruit films to stimulate saliva secretion (16). Fiber intake can also have adverse effects, such as bloating, diarrhea, or constipation, so it is recommended to gradually increase the dose during intake to establish gastrointestinal tolerance (61, 62).

It should be admitted that this meta-analysis has several limitations. Firstly, our study did not separate male and female patients with diabetes, so we could not observe the differences in the control of sticky soluble dietary fiber in blood glucose and blood lipid between these two groups. Secondly, the longest study period among the included studies was only 16 weeks, since only a few studies with a longer period met the inclusion criteria, and only one article had an intervention period of 52 weeks (36). To maintain consistency in the intervention period, we chose results from the first 16 weeks of the study. Longer intervention cycles should be considered and higher quality RCTs should be conducted to better obtain the long-term efficacy of sticky soluble dietary fiber. Besides, the number of studies on some types of dietary fiber was significantly limited. For example, only 2 studies on glucomannan and β-glucan and only 1 study on Cassia tora were included in this study, whereas 6 to 7 studies on guar gum and psyllium were included. More studies on these fiber types should be conducted in subsequent research. In addition, the type of medication during the trial may also impact the effect of the viscous fiber intervention. However, due to the limited information provided by each trial, we did not conduct a more detailed analysis to identify the potential impact of the medication. Finally, the included studies were highly heterogeneous. Therefore, more RCTs with a large number of participants and more reasonable designs are required.

## 5. Conclusion

This meta-analysis confirmed that the supplementation of viscous soluble dietary fiber has potential benefits for the control of blood glucose and lipids in patients with T2DM. In addition, the recommended supplemental dose is from 8.3 g to 10.2 g/day, and the recommended duration of supplemental treatment is more than 6 weeks.

## Data availability statement

The original contributions presented in the study are included in the article/Supplementary material, further inquiries can be directed to the corresponding author.

## Author contributions

KL: Conceptualization, Data curation, Formal analysis, Investigation, Methodology, Validation, Visualization, Writing—original draft. TY: Writing—original draft, Data curation, Investigation. XC: Writing—original draft, Data curation, Investigation. HX: Writing—review and editing. SW: Writing—review and editing. GS: Writing—review and editing. LC: Writing—review and editing. WL: Funding acquisition, Methodology, Writing—original draft, Conceptualization.
