# Supplementary material for: Effect of viscous soluble dietary fiber on glucose and lipid metabolism in patients with type 2 diabetes mellitus: a systematic review and meta-analysis on randomized clinical trials
Source: Front Nutr. 2023 Aug 31;10:1253312. doi: 10.3389/fnut.2023.1253312 (PMC10500602; doi:10.3389/fnut.2023.1253312)
Supplement: Supplementary file 1 [file Data_Sheet_1.docx]

Supplementary Material

# Supplementary Tables

**Supplementary Table 1.** Baseline characteristics of the included studies.

| Author | Year | Study type | Country | Sample capacity | M^a^ | F^b^ | Ages（years） | BMI（kg/m^2^） | Duration of diabetes (years) | Intervention  fiber type | Control material | Dose（g/day） | Duration of intervention（weeks） |
| --- | --- | --- | --- | --- | --- | --- | --- | --- | --- | --- | --- | --- | --- |
| Anderson | 1999 | parallel | America | 29 | 29 | 0 | I^c^:62; C^d^:63.8 | I:28.7; C:27.4 | stable T2DM^e^ | psyllium | microcrystalline cellulose | 10.2 | 8 |
| Aro | 1981 | cross-over | Finland | 9 | 5 | 4 | 53 | NA | 6.5 | guar gum | placebo | 21 | 12 |
| Abutair | 2016 | parallel | Palestine | 36 | NA | NA | 35-60 | I:31.8; C:31.5 | newly identified (≤1year) | psyllium | without psyllium | 10.5 | 8 |
| Chen | 2013 | cross-over | China | 22 | 10 | 12 | 64.2 | 25.5 | ≥1year | Konjac Glucomannan | placebo | 3.6 | 4 |
| Cho | 2005 | parallel | Korea | 30 | I:7; C:7 | I:8; C:8 | 57.1 | 23.7 | 10.8 | Cassia tora | placebo（maltodextrin） | 4 | 8 |
| Christine | 2009 | parallel | France | 53 | 32 | 21 | I:61.9; C:61.8 | I:30.48; C:29.02 | 1-15 | β-glucan | without β-glucan | 3.5 | 8 |
| Feinglos-1 | 2013 | parallel | America | 23 | I:10; C:6; | I:3; C:2; | I:61.8; C:56.5; | NA | ≥1year | psyllium | placebo | 6.8 | 12 |
| Feinglos-2 | 2013 | parallel | America | 22 | I:9; C:6 | I:3; C:2 | I:64.8; C:56.5 | NA | ≥1year | psyllium | placebo | 13.6 | 12 |
| Fuessl | 1986 | cross-over | The UK | 18 | 12 | 6 | 61.3 | 30.1 | 7.1 | guar gum | placebo | 12.7 | 4 |
| Ghalandari | 2017 | parallel | Iran | 34 | I:5; C:6 | I:13; C:10 | I:55.9; C:54.2 | I:28.5; C:30.3 | I:8.4; C:7.4 | psyllium | placebo (corn starch) | 3.1 | 8 |
| Lalor | 1989 | cross-over | The UK | 19 | 8 | 11 | 58 | 31.5 | NA | guar gum | placebo | 15 | 12 |
| Liatis | 2009 | parallel | Greece | 41 | I:12; C:11 | I:11; C:7 | I:60.22; C:66.50 | I:29.61; C:27.01 | I:7.6; C:10.1 | betaglucan | without betaglucan | 3 | 3 |
| Niemi | 1988 | cross-over | Finland | 22 | 16 | 6 | 63 | 27 | NA | guar gum | microcrystalline cellulose | 15 | 12 |
| Peterson-1 | 1987 | cross-over | The UK | 16 | 10 | 6 | 60 | 27.3 | 9 | guar gum | without guar gum | 7.6 | 6 |
| Peterson-2 | 1987 | cross-over | The UK | 16 | 10 | 6 | 60 | 27.3 | 9 | guar gum | without guar gum | 8.3 | 6 |
| Reimer | 2020 | parallel | Canada | 166 | NA | NA | I:56.2;  C:53.4 | I:39.5; C:41.0 | stable T2DM | PGX^f^ | placebo (rice flour） | 15-20 | 16 |
| Uusitupa | 1989 | parallel | Finland | 39 | I:8; C:5 | I:12; C:14 | I:M58,F61.5; C:M59.6,F61.4 | NA | I:M11.3,F8.6; C:M8.6,F9.2 | guar gum | placebo (wheat flour) | 15 | 12 |
| Vuksan | 1999 | cross-over | Italy | 11 | 5 | 6 | M:62; F:59 | NA | M:11.5; F:18.1 | glucomannan | placebo（wheat bran） | 12.8 | 3 |
| Ziai | 2005 | parallel | Iran | 36 | NA | NA | I:51.9; C:53.6 | I:26.6; C:27.5 | NA | psyllium | microcrystalline cellulose placebo | 10.2 | 8 |

a: M = male; b: F = female. c: I = [Intervention group](javascript:;) d: C = Control group. e: T2DM = Type 2 diabetes mellitus. f: PGX: active ingredient: (α-D-glucurono-α-D-manno-β-D-manno-β-D-gluco), (α-Lglucurono-β-D-mannurono), β-D-gluco-β-D-mannan.

**Supplementary Table 2:** Subgroup analysis results of the included studies.

| **Index** | **Subgroup** | **No. of trials** | **Mean difference** | | |  | **Heterogeneity** | |
| --- | --- | --- | --- | --- | --- | --- | --- | --- |
|  |  |  | **Mean** | **95%CI** | **p^a^** |  | **I^2^(%)** | **p^b^** |
| **HbA1c** | **Region** |  |  |  |  |  |  |  |
|  | North America | 4 | -0.40 | (-0.58, -0.21) | < 0.001 |  | 42.5 | 0.156 |
|  | Asia | 3 | -1.24 | (-2.35, -0.14) | 0.028 |  | 71.9 | 0.028 |
|  | Europe | 7 | -0.22 | (-0.42, -0.02) | 0.031 |  | 0.0 | 0.712 |
|  | **Study type** |  |  |  |  |  |  |  |
|  | parallel | 10 | -0.45 | (-0.66, -0.23) | < 0.001 |  | 68.6 | 0.001 |
|  | cross-over | 4 | -0.85 | (-1.58, -0.12) | 0.022 |  | 0.0 | 0.963 |
|  | **Fiber type** |  |  |  |  |  |  |  |
|  | β-glucan | 2 | -0.16 | (-0.37, 0.06) | 0.152 |  | 0.0 | 0.934 |
|  | psyllium | 5 | -0.72 | (-1.08, -0.37) | < 0.001 |  | 78.7 | 0.001 |
|  | Cassia tora | 1 | -0.30 | (-1.44, 0.84) | 0.607 |  | / | / |
|  | guar gum | 5 | -0.70 | (-1.28, -0.12) | 0.018 |  | 0.0 | 0.947 |
|  | PGX | 1 | -0.08 | (-0.42, 0.26) | 0.649 |  | / | / |
|  | **Dosage of viscous soluble dietary fiber** | | | |  |  |  |  |
|  | ≤ 8.3g/day | 6 | -0.25 | (-0.43, -0.07) | 0.008 |  | 0.0 | 0.706 |
|  | > 8.3g/day | 8 | -0.64 | (-0.95, -0.32) | < 0.001 |  | 71.2 | 0.001 |
|  | **Duration of intervention** | | |  |  |  |  |  |
|  | ≤ 6weeks | 4 | -0.19 | (-0.43, 0.05) | 0.121 |  | 0.0 | 0.679 |
|  | > 6weeks | 10 | -0.52 | (-0.76, -0.28) | < 0.001 |  | 65.1 | 0.002 |
| **FBG** | **Region** |  |  |  |  |  |  |  |
|  | North America | 4 | -0.94 | (-1.83, -0.05) | 0.038 |  | 81.7 | 0.001 |
|  | Asia | 5 | -1.21 | (-3.23, 0.80) | 0.238 |  | 91.9 | < 0.001 |
|  | Europe | 10 | -0.76 | (-1.22, -0.29) | 0.001 |  | 0.0 | 0.907 |
|  | **Study type** |  |  |  |  |  |  |  |
|  | parallel | 11 | -0.88 | (-1.55, -0.20) | 0.001 |  | 84.9 | < 0.001 |
|  | cross-over | 8 | -1.20 | (-1.91, -0.50) | 0.001 |  | 6.4 | 0.381 |
|  | **Fiber type** |  |  |  |  |  |  |  |
|  | β-glucan | 2 | -0.66 | (-1.27, -0.05) | 0.033 |  | 0.0 | 0.951 |
|  | psyllium | 6 | -1.40 | (-2.50, -0.31) | 0.012 |  | 89.7 | < 0.001 |
|  | glucomannan | 2 | -1.82 | (-3.25, -0.40) | 0.012 |  | 27.2 | 0.241 |
|  | Cassia tora | 1 | 1.17 | (-1.18, 3.52) | 0.328 |  | / | / |
|  | guar gum | 7 | -0.91 | (-1.67, -0.15) | 0.020 |  | 0.0 | 0.701 |
|  | PGX | 1 | 0.25 | (-0.38, 0.88) | 0.439 |  | / | / |
|  | **Dosage of viscous soluble dietary fiber** | | | |  |  |  |  |
|  | ≤ 8.3g/day | 8 | -0.44 | (-1.41, 0.52) | 0.370 |  | 75.3 | < 0.001 |
|  | > 8.3g/day | 11 | -1.29 | (-1.97, -0.61) | < 0.001 |  | 76.4 | < 0.001 |
|  | **Duration of intervention** | | |  |  |  |  |  |
|  | ≤ 6weeks | 6 | -1.00 | (-1.62, -0.37) | 0.002 |  | 14.0 | 0.325 |
|  | > 6weeks | 13 | -0.93 | (-1.63, -0.24) | 0.008 |  | 82.6 | < 0.001 |
| **Fasting** | **Region** |  |  |  |  |  |  |  |
| **insulin** | Asia | 3 | -4.39 | (-10.13, 1.35) | 0.134 |  | 94.3 | < 0.001 |
|  | Europe | 4 | -2.60 | (-5.30, 0.09) | 0.059 |  | 7.0 | 0.358 |
|  | **Study type** |  |  |  |  |  |  |  |
|  | parallel | 4 | -4.93 | (-9.84, -0.02) | 0.049 |  | 92.0 | < 0.001 |
|  | cross-over | 3 | -1.38 | (-4.26, 1.49) | 0.345 |  | 0.0 | 0.923 |
|  | **Fiber type** |  |  |  |  |  |  |  |
|  | β-glucan | 1 | -7.00 | (-12.59, -1.41) | 0.014 |  | / | / |
|  | psyllium | 3 | -4.39 | (-10.13, 1.35) | 0.134 |  | 94.3 | < 0.001 |
|  | glucomannan | 1 | 0.34 | (-15.30, 15.98) | 0.966 |  | / | / |
|  | guar gum | 2 | -1.44 | (-4.37, 1.48) | 0.333 |  | 0.0 | 0.739 |
|  | **Dosage of viscous soluble dietary fiber** | | | |  |  |  |  |
|  | ≤ 10.2g/day | 5 | -1.49 | (-2.62, -0.36) | 0.010 |  | 2.2 | 0.394 |
|  | > 10.2g/day | 2 | -8.11 | (-18.29, 2.07) | 0.118 |  | 51.1 | 0.153 |
|  | **Duration of intervention** | | |  |  |  |  |  |
|  | ≤ 6weeks | 4 | -2.60 | (-5.30, 0.09) | 0.059 |  | 7.0 | 0.358 |
|  | > 6weeks | 3 | -4.39 | (-10.13, 1.35) | 0.134 |  | 94.3 | < 0.001 |

CI: Confidence interval. PGX: active ingredient: (α-D-glucurono-α-D-manno-β-D-manno-β-D-gluco), (α-Lglucurono-β-D-man nurono), β-D-gluco-β-D-mannan. HbA1c: glycosylated hemoglobin. FBG: fasting blood-glucose.

a: p-values of mean difference; b: p-values for heterogeneity between subgroups.

**Supplementary Table 3.** Sensitivity analysis: Specific changes in overall heterogeneity and effect variables after the deletion of one or two studies.

| **Index** | **Deletion study** | **Heterogeneity before removal of a study** | |  | **Heterogeneity after removal of a study** | |  | **Mean difference before removal of a study** | | |  | **Mean difference after removal of a study** | | |
| --- | --- | --- | --- | --- | --- | --- | --- | --- | --- | --- | --- | --- | --- | --- |
|  |  | **I^2^(%)** | **p** |  | **I^2^(%)** | **p** |  | **Mean** | **95%CI** | **p** |  | **Mean** | **95%CI** | **p** |
| HbA1c | Abutair 2016 and Ziai 2005 | 57 | 0.004 |  | 4 | 0.40 |  | -0.47 | (-0.66, -0.27) | < 0.00001 |  | -0.36 | (-0.46, -0.26) | < 0.00001 |
| fasting insulin | Abutair 2016 | 84 | < 0.00001 |  | 0 | 0.53 |  | -3.64 | (-6.98, -0.30) | < 0.00001 |  | -1.47 | (-2.55, -0.39) | 0.008 |
| TC | Christine 2009 | 19 | 0.25 |  | 0 | 0.46 |  | -0.33 | (-0.46, -0.21) | < 0.00001 |  | -0.40 | (-0.54, -0.26) | < 0.00001 |
| TC | Ziai 2005 | 19 | 0.25 |  | 5 | 0.40 |  | -0.33 | (-0.46, -0.21) | < 0.00001 |  | -0.35 | (-0.48, -0.22) | < 0.00001 |
| LDL-C | Chen 2013 and Christine 2009 | 37 | 0.09 |  | 0 | 0.48 |  | -0.24 | (-0.35, -0.13) | < 0.0001 |  | -0.26 | (-0.38, -0.13) | < 0.0001 |
| HDL-C | Ziai2005 | 26 | 0.18 |  | 0 | 1.00 |  | 0.02 | (-0.02, 0.06) | 0.37 |  | 0.00 | (-0.04, 0.04) | 0.88 |

HbA1c = glycosylated hemoglobin; TC = total cholesterol; LDL-C = low density lipoprotein cholesterol; HDL-C = high density lipoprotein cholesterol.

# Supplementary Figure

**
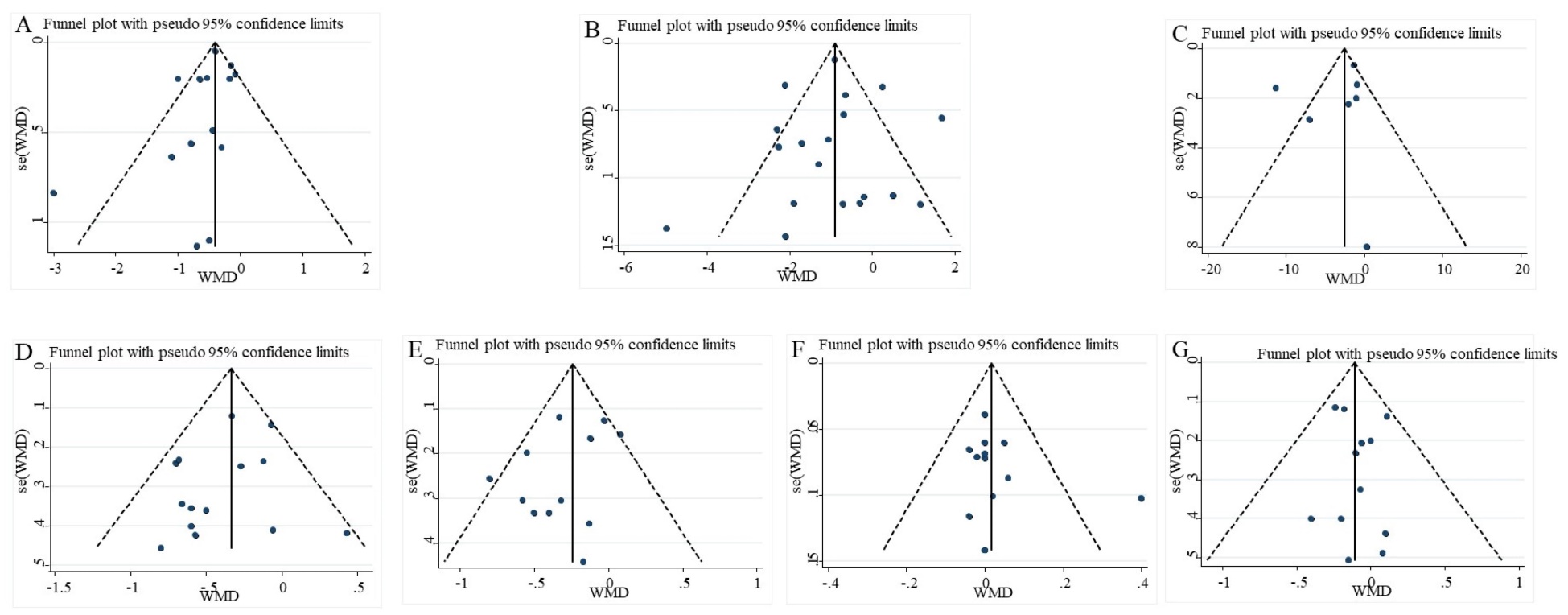
**

**Supplementary Figure.** Funnel plots were used to assess the publication bias of sticky soluble dietary fiber for HbA1c (A), FBG (B), fasting insulin (C), TC (D), LDL-C (E), HDL-C (F), and TG (G). HbA1c = glycosylated hemoglobin; FBG = fasting blood-glucose; TC = total cholesterol; LDL-C = low density lipoprotein cholesterol; HDL-C = high density lipoprotein cholesterol; TG = triglyceride.
